# A high-quality reference genome for the fish pathogen *Streptococcus iniae*

**DOI:** 10.1099/mgen.0.000777

**Published:** 2022-03-01

**Authors:** Areej S. Alsheikh-Hussain, Nouri L. Ben Zakour, Brian M. Forde, Oleksandra Rudenko, Andrew C. Barnes, Scott A. Beatson

**Affiliations:** ^1^​ School of Chemistry & Molecular Biosciences, The University of Queensland, Brisbane, Queensland, Australia; ^2^​ Australian Infectious Diseases Research Centre, The University of Queensland, Brisbane, Queensland, Australia; ^3^​ The Westmead Institute for Medical Research and the University of Sydney, Sydney, New South Wales, Australia; ^4^​ School of Biological Science, The University of Queensland, Brisbane, Queensland, Australia

**Keywords:** SMRT sequencing, insertion sequence, reference-guided assembly, misassembly, mobile genetic elements

## Abstract

Fish mortality caused by *Streptococcus iniae* is a major economic problem in aquaculture in warm and temperate regions globally. There is also risk of zoonotic infection by *S. iniae* through handling of contaminated fish. In this study, we present the complete genome sequence of *S. iniae* strain QMA0248, isolated from farmed barramundi in South Australia. The 2.12 Mb genome of *S. iniae* QMA0248 carries a 32 kb prophage, a 12 kb genomic island and 92 discrete insertion sequence (IS) elements. These include nine novel IS types that belong mostly to the IS*3* family. Comparative and phylogenetic analysis between *S. iniae* QMA0248 and publicly available complete *S. iniae* genomes revealed discrepancies that are probably due to misassembly in the genomes of isolates ISET0901 and ISNO. Long-range PCR confirmed five rRNA loci in the PacBio assembly of QMA0248, and, unlike *S. iniae* 89353, no tandemly repeated rRNA loci in the consensus genome. However, we found sequence read evidence that the tandem rRNA repeat existed within a subpopulation of the original QMA0248 culture. Subsequent nanopore sequencing revealed that the tandem rRNA repeat was the most prevalent genotype, suggesting that there is selective pressure to maintain fewer rRNA copies under uncertain laboratory conditions. Our study not only highlights assembly problems in existing genomes, but provides a high-quality reference genome for *S. iniae* QMA0248, including manually curated mobile genetic elements, that will assist future *S. iniae* comparative genomic and evolutionary studies.

## Impact Statement

Here we describe a high-quality, complete PacBio/ONT/Illumina genome for the aquatic pathogen *Streptococcus iniae *. Manual curation of its mobile genetic element (MGE) content, including a genomic island, prophage and 92 insertion sequences (IS), identified several novel IS and resulting pseudogenes. We also present the first analysis of the *S. iniae* methylome. Comparative and phylogenetic analysis between *S. iniae* QMA0248 and publicly available complete *S. iniae* genomes revealed discrepancies that are probably due to misassembly in the genomes of strains ISET0901 and ISNO. We also characterized differences in rRNA copy number between *S. iniae* genomes and found evidence that QMA0248 does harbour a tandem repeat rRNA loci similar to *S. iniae* 89353, but it appears to be unstable under laboratory conditions and there is probably a selective advantage for its loss under some laboratory conditions. Our study not only highlights assembly problems in existing *S. iniae* genomes, but also provides a high-quality reference genome for *S. iniae* QMA0248, including manually curated MGEs, that will assist future *S. iniae* comparative genomic and evolutionary studies.

## Data Summary

Sequence data including Oxford Nanopore Technologies (ONT) MinION reads, Pacific Biosciences (PacBio) RSII reads, genome assembly and methylation motif summary for *S. iniae* QMA0248 have been submitted to the National Center for Biotechnology Information (https://www.ncbi.nlm.nih.gov) under the BioProject Accession PRJNA385746 and GenBank Accession CP022392. Illumina reads for QMA0248 are available under the Bioproject PRJNA417543 (SRA Accession SRX4071375).

## Introduction

*Streptococcus iniae* is a pathogen that causes mortality in a wide range of fish species in wild and farmed, marine and freshwater environments, resulting in large economic losses to aquaculture [1, 2]. *S. iniae* is also considered an opportunistic human pathogen, causing sporadic infections mostly in the elderly who have more than one underlying health condition, such as diabetes mellitus or chronic rheumatic heart disease [3, 4]. *S. iniae* pathogenesis is imparted through a repertoire of virulence factors (VFs) including surface proteins, secreted toxins and capsular polysaccharide (CPS) [4]. VFs can be acquired through lateral gene transfer (LGT) of mobile genetic elements (MGEs) such as composite transposons, genomic islands (GIs) or prophages.

MGEs are a means by which bacterial pathogens acquire traits that help adapt to changing conditions including vaccination, antibiotics, a new host or new environment [5, 6]. Indeed, they are considered the main drivers of gene flux in bacteria, contributing to diversity within species [7]. Insertion sequence (IS) elements, for instance, are small MGEs (0.7–3.5 kb) that have an important role in evolution and genome plasticity. IS insertion within bacterial chromosomes or plasmids can result in genetic modifications through insertional inactivation of genes or up-regulation of adjacent intact genes through outward-facing promoter sequences carried by some IS [8, 9]. In some cases, pairs of IS can mobilize intervening sequence as a composite transposon [8]. The mobility of IS elements leads to their expansion or loss within bacterial lineages. Expansion is associated with accumulation of pseudogenes, which is considered an early stage in genome reduction as a mechanism for adaptation [9]. Accordingly, to obtain the complete evolutionary picture within bacterial species it is important to study the distribution and abundance of IS elements.

As yet no study has focused on the diversity and distribution of MGEs in *S. iniae* genomes. In fact, only four complete *S. iniae* genomes were deposited in GenBank at the commencement of the present study, none of which had comprehensive annotations for MGEs: SF1, YSFST01-82, ISET0901 and ISNO. *S. iniae* SF1 (accession: CP005941) was cultured from moribund flounder from a fish farm that experienced an epidemic in North China [10]. YSFST01-82 (accession: CP010783) is an isolate from a diseased olive flounder from South Korea (2012) [11]. ISET0901 (accession: CP007586) is reported to be a highly virulent strain, which was isolated from diseased Nile tilapia during an outbreak in Israel (2005) [12]. Strain ISNO (accession: CP007587) was obtained through selection for resistance of *S. iniae* ISET0901 to novobiocin [13].

There are currently 25 different IS families in the ISFinder database, but only one IS is described for *S. iniae* (IS*Stin1* of the family IS*256*). As is typical for complete bacterial genomes, the annotations of the four complete *S. iniae* genomes available at GenBank are limited to some of the transposase genes associated with IS elements without definition of the IS boundaries. Indeed, the difficulty in annotating IS elements and the lack of reliable automated annotation methods means that only a small subset of complete genomes have accurate IS annotations. Small partial IS elements are often disregarded although they provide valuable insights into ancestral recombination events. With the increasing availability of long-read sequencing there is a need for a high-quality, well-characterized *S. iniae* reference genome that better enables the impact of IS elements on evolution and diversity to be determined.

In this study, we have completely characterized the genome of *S. iniae* QMA0248. Manual curation of annotations for IS, genomic islands, prophages and CRISPR was carried out along with a comparison with the four publicly available complete genomes from NCBI (SF1, YSFST01-82, ISET0901 and ISNO). Comparative and phylogenetic analyses revealed discrepancies between the MGE content, indicating probable misassembly in the genome of ISNO and ISET0901. The complete genome of QMA0248 will provide an important scaffold for future phylogenomic studies of *S. iniae *.

## Methods

### Bacterial strain and sequencing

*S. iniae* strain QMA0248 was isolated from diseased barramundi (*Lates calcarifer*) from a farm in South Australia in 2009 [14]. Genomic DNA was prepared from several well-isolated colonies of *S. iniae* QMA0248 grown for 18 h on Todd–Hewitt agar from a master seed stock (non-subcultured) with the Genomic Tip 20 kit (Qiagen). Pre-incubation for 2 h at 37 °C of cells suspended in 500 µl of 50 mM EDTA containing 200 units of mutanolysin and 2 mg lysozyme was found to improve cell lysis prior to following the manufacturer’s protocol for purification of high-molecular-weight DNA. The genome of strain QMA0248 was sequenced using three SMRT cells on the Pacific Biosciences (PacBio) RS II platform and P4C2 sequencing chemistry, which generated a total of 57083 reads with an average length of 6178 bp. Reads were deposited at BioProject PRJNA385746 under accession SRP109617. The genome of strain QMA0248 was also sequenced using Illumina Nextera libraries on a HiSeq2000.

### Genome assembly and detection of modified bases

PacBio sequencing reads derived from *S. iniae* QMA0248 genomic DNA were assembled using HGAP (hierarchical genome assembly process) using the PacBio Single Molecule Real Time (SMRT) Portal (v2.3.0) [15], with default settings and minimum seed read length of 4509 bp. Contiguity was used to visualize the assembly and the overlap between contigs using BLASTn [16, 17]. The resulting assembly was used as a reference in the RS Resequencing module of PacBio’s SMRT Analysis v2.3.0 to map the raw reads onto the reference genome, producing a highly accurate genome consensus. Illumina-sequenced reads of strain QMA0248 were mapped to the PacBio assembly using Snippy v3.0 (https://github.com/tseemann/snippy). To analyse read pileup in the potential rRNA tandem operon, raw reads of QMA0248 were mapped onto the ~7 kb rRNA contig using BLASR v2.2, as part of PacBio’s SMRT Analysis Suite, and visualized using Artemis [18]. Methylated DNA bases were identified in the resulting genome assembly using the RS Modification and Motif Analysis protocol and Motif Finder v1 within the SMRT Analysis suite v2.3.0 using a Quality Value (QV) cutoff of 30. DNA methyltransferases (MTases) were identified using nucleotide comparisons against REBASE [19].

### ONT MinION sequencing to resolve tandem rRNA repeats

To fully resolve the tandem or single arrangement of the locus, additional long read sequencing was performed using the Oxford Nanopore Technologies MinION instrument. Briefly, genomic DNA was extracted from QMA0248 cells recovered directly from overnight cultures on Columbia agar containing 5 % defibrinated sheep blood (Oxoid). Cells were suspended and washed in 200 µl sterile nuclease-free water and high-molecular-weight DNA was extracted using the cetyl-trimethylammonium bromide (CTAB) method [20] and quantified by Qubit fluorimetry (Thermo). DNA (1 µg) was prepared for sequencing using the ligation sequencing kit (SQK-LSK109) and native barcoding kit (NBD104) following the manufacturer’s protocol (Oxford Nanopore Technologies). The library was multiplexed with 11 other libraries and sequenced for 40 h on a Minion Flow Cell (version R9.4.1). Raw reads have been deposited in the sequence read archive at NCBI under accession SRX9700218.

Raw nanopore reads were demultiplexed and barcodes were removed with porechop 0.2.3 (https://github.com/rrwick/Porechop). For draft assembly and closure of the QMA0248 genome, reads were randomly subsampled to an estimated 250× coverage and assembled with Flye 2.7-b1585 [21]. Assembly graphs were inspected with bandage 0.8.1 [22] and revealed closure and circularization of the genome. To correct base-calling errors in the nanopore data and resolve indels and homopolymers, the initial assembly was polished iteratively using the unicycler polish tool, including likelihood-based assessment of each round with ALE [23]. The resulting polished assembly of QMA0248 was reoriented to position the origin of chromosomal replication to 0 with the fixstart tool in circlator 1.5.5 [24] and contained a tandem repeat in the first rRNA locus of the chromosome.

As there was evidence for both single and tandem repeats at this locus, we filtered the raw nanopore reads to retain those >8 kb and therefore capable of spanning both single and tandem repeat of the locus and mapped to both the original Pacbio assembly with the single copy and the new Nanopore-based assembly with the tandem repeat using minimap2 [25]. The resulting sam files were converted to bam and indexed using samtools and then viewed using Artemis v18.1.0.

### PCR to investigate rRNA tandem duplication

To investigate the presence of an rRNA tandem repeat, long-range PCR from a unique adjacent region to a unique adjacent region was performed using specific primers for each of five rRNA regions (Table S6, available in the online version of this article), which were designed using Primer3 [26]. PCR was done using a LongAmp Taq PCR Kit (NEB) from 40 ng of QMA0248 *S. iniae* genomic DNA as follows: 5 min at 94 °C; 30 cycles of 30 s at 94 °C, 30 s at 56 °C and 15 min at 65 °C; and 10 min final extension at 65 °C. The gel was loaded with 5 µl QUICK-LOAD 1 kb Extend DNA Ladder and 1 µl of PCR products and run for 90 min at 70 V using 0.7 % TAE buffer solution and stained with HydraGreen. Following the finding that the majority of spanning reads in the ONT assembly supported the rRNA tandem repeat, PCRs were carried out under previously used conditions except that DNA extracted using the CTAB method was provided as a template (the extraction used for ONT library preparation).

### Annotation of genome and mobile genetic elements

Automated genome annotation was done using Prokka v1.11 (Prokaryotic Genome Annotation System) [27] and then manually curated using Artemis [18] and Geneprimp [28]. The start codons of known coding sequences (CDS), such as in the capsule and streptolysin S operons, were further adjusted where appropriate using UniProtKB (http://www.uniprot.org/) and Pfam [29]. CRISPRs (clustered regularly interspaced short palindromic repeats) were predicted using the CRISPR finder tool (http://crispr.u-psud.fr/crispr/) [30]. Prophage annotation was done using PHAST (Phage Search Tool) (http://phast.wishartlab.com/) [31]. Island Viewer (http://www.pathogenomics.sfu.ca/islandviewer/) was used to predict GIs [32]. Boundaries of phage and GI regions were manually adjusted to their respective attachment sites. IS Saga (http://www-genome.biotoul.fr/) was used for the initial identification of IS. Additional manual curation was carried out to confirm the boundaries of complete and partial IS elements using the IS Finder database (http://www-is.biotoul.fr/). IS element matches against the database that showed ≥95 % nucleotide identity were assigned the top matching IS name. IS elements of lower identity are novel and were assigned the names IS*Stin2*–IS*Stin10* by IS Finder. The impact of IS elements on flanking coding sequences was analysed using Artemis and by searching the amino acid sequence in UniProt KB (http://www.uniprot.org/) and Pfam databases [18, 29]. The complete annotated genome sequence was deposited at GenBank under accession number CP022392.

### Comparative genomics and phylogenetic analysis

Alignments of the whole-genome or genomic sub-regions, such as the CRISPR and GIs, were done using BLASTn implemented in EasyFig v2.1 [17, 33]. Detailed analysis of regions of difference and comparison of IS were done using the Artemis Comparison Tool (ACT) [34]. The core genome of the five genomes QMA0248, SF1, YSFST01-82, ISET0901 and ISNO was defined using Roary [35]. Phylogenetic trees were reconstructed using the core genome and the core SNP methods, using multiple programs (see below), using strain QMA0140 as the out-group [36]. *S. iniae* QMA0140 is a dolphin isolate from the USA in 1976, and was sequenced using Illumina HiSeq 2000 (see Bioproject PRJNA417543; SRA Accession SRX4071375). For quality control, the first 20 bp of each read derived from QMA0140 genomic DNA was hard trimmed using Nesoni v0.132 (https://github.com/Victorian-Bioinformatics-Consortium/nesoni) with a minimum length and quality of 70 and 20, respectively. Hard trimmed filtered reads of QMA0140 were assembled using SPAdes v3.9.0 where contigs with <10× coverage and smaller than 100 bp were removed [37]. For core genome phylogenies, whole-genome alignment of the five complete genomes and QMA0140 draft assembly was done using Mauve v2.4.0 and Parsnp v1.2 with default parameter settings [38, 39]. For the alignment using Mauve, conserved blocks in all six genomes longer than 500 bp were selected and concatenated using the stripSubsetLCBs script, producing the core genome alignment. For core SNP phylogenies, error-free simulated reads were created using wgsim v0.3.2 (https://github.com/lh3/wgsim) and mapped to the reference genome QMA0248 along with QMA0140 hard trimmed and filtered raw reads using bowtie v1.0.0 [40], where variants were called using Nesoni v0.132 (https://github.com/Victorian-Bioinformatics-Consortium/nesoni) using default parameters. Core SNPs were also identified by mapping the reads of the six genomes to QMA0248 using BWA-MEM v0.7.15 (r1140), implemented in Snippy v3.0 (https://github.com/tseemann/snippy) [41]. All trees were produced by RAxML v8.2.9 [42] using the general time-reversible (GTR) and GAMMA distribution model of among-site rate variation with bootstrapping from 1000 replicates, and were viewed using FigTree v1.4.0 (http://tree.bio.ed.ac.uk/software/figtree).

## Results and discussion

### Genomic features of *S. iniae* QMA0248

**Fig. 1.**
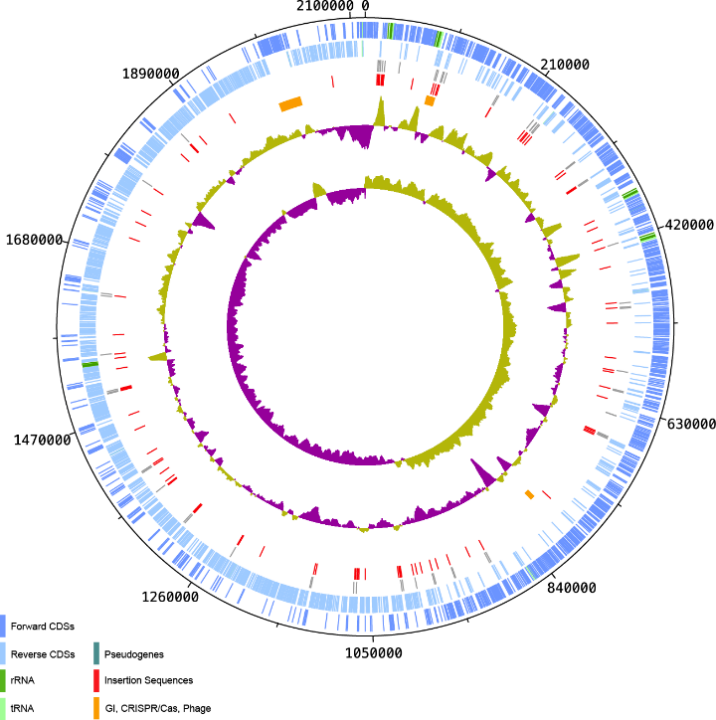
Circular map of the *S. iniae* QMA0248 genome. Genomic features from outer ring to inner ring are described in the key to the left, where the innermost two rings correspond to the GC skew (inner) and GC plot (outer). CDS: coding sequence. GI: genomic island. The circular map was generated using DNAPlotter [70].

The genome of *S. iniae* QMA0248 consists of a single circular chromosome of 2 116 570 bp with no plasmids (Fig. 1, Table 1). The QMA0248 chromosome has an average GC content of 36.8 %, consistent with the other four *S. iniae* genomes (SF1, YSFST01-82, ISET0901 and ISNO) and in common with several other *Streptococcus* species such as *S. agalactiae* (35.6 %) [43], *S. pneumoniae* (39.7 %) [44] and *S. pyogenes* (38.5 %) [45]. Of note, there is a high degree of strand bias in the genome of *S. iniae* QMA0248, where genes are preferentially orientated in the leading strand, which is typical for *Firmicutes* (Fig. 1). Only 7 % of QMA0248 protein-coding genes are annotated as hypothetical proteins compared with a quarter in SF1 and 11–14 % in the other three published genomes: YSFST01-82, ISET0901 and ISNO. There are 68 pseudogenes identified in QMA0248 (GenBank assembly accession: CP022392.1), most due to interruption by IS (Fig. 1, Table S1). This is approximately five times greater than the number of pseudogenes predicted in SF1. Collectively, these differences between the compared genomes QMA0248, SF1, YSFST01-82, ISET0901 and ISNO are likely to reflect different approaches to annotation. The other 14 pseudogenes are caused by in-frame stop codons or frame shifts, all supported by additional mapping of Illumina reads of strain QMA0248 against its PacBio assembly.

**Table 1.** General features of five *S. iniae* complete genomes

Feature	QMA0248	SF1	YSFST01-82	ISET0901	ISNO
Accession number	CP022392	CP005941	CP010783	CP007586	CP007587
Genome size (bp)	2 116 570	2 149 844	2 086 959	2 070 822	2 070 182
GC content (%)	36.8	36.7	36.8	36.8	36.8
Total CDS number	1946	2125	1897	1872	1865
Total gene number	2196	2196	2029	1997	1996
rRNAs (5S, 16S, 23S)	15	12	15	12	12
tRNAs	58	45	58	45	45
Reference	This study	[12]	[11]	[12]	[13]
Assembly type	PacBio RSII P2C4	454 FLX+/Illumina MiSeq/Sanger	454 FLX Titanium/Opgen/Sanger	Illumina 1500 HiSeq/Reference guided assembly	Illumina 1500 HiSeq/Reference guided assembly

QMA0248 has a single CRISPR locus which harbours a tandem array of 15 identical 36 bp repeats, separated by 14 distinct 30 bp spacers, which is about double the size of the CRISPR array in SF1, ISET0901 and ISNO (Fig. S1). Upstream of QMA0248 CRISPR are four Cas genes, *csn2*, *cas2*, *cas1* and *cas9*, which alongside the CRISPR locus provide adaptive immunity against foreign DNA (e.g. phage and plasmids) [46].

QMA0248 harbours 58 tRNA genes and 15 rRNA loci, consisting of 5S, 16S and 23S genes, arranged in five loci. In contrast, there is one rRNA operon fewer in SF1, ISET0901 and ISNO than in QMA0248 (Table 1). Furthermore, during the preparation of this paper, a PacBio complete genome was published for *S. iniae* 89 353 (accession: CP017952), which has an identical rRNA arrangement to QMA0248 except that one rRNA locus encodes an ~7 kb tandem duplication (i.e. six loci in total) [47]. Such intraspecies variation in the number of rRNA genes (and tRNA genes) is not uncommon in bacteria (including streptococci) [48, 49], and prompted us to investigate further.

### Investigation of rRNA assembly in the *S. iniae* QMA0248 genome

PacBio reads for QMA0248 were initially assembled into a large ~2 Mb contig representing most of the chromosome of *S. iniae* QMA0248 and three contigs <10 kb in length. The short contigs appeared to be single-read chimaeras that were discarded from the final assembly. However, the identification of the tandem rRNA region in *S. iniae* 89 353 prompted us to review the assembled short contigs. One of these ~7 kb discarded contigs encoded an rRNA region (5S, 16S and 23S genes in tandem with an intervening cluster of tRNA genes). Subsequent reassembly and visualization of mapped raw reads indicated that the additional rRNA contig could be placed in three of the five rRNA operon locations to form an ~13 kb putative tandem duplication of 5S, 16S and 23 rRNA genes as seen in the 89 353 genome [47]. Closer examination of the read pileup for the ~7 kb rRNA contig revealed that the tandem duplication was not well supported by overlapping reads (Fig. S2), suggesting that it may be a chimeric assembly of reads from more than one rRNA locus. To investigate the location of the putative tandem rRNA duplication in *S. iniae* QMA0248, we carried out long-range PCR across each of the five potential rRNA loci in the chromosome. PCR revealed no tandem rRNA duplication in any locus (Fig. S3). These results led us to speculate that during the culturing step, prior to DNA extraction and genome sequencing, there existed a subpopulation of QMA0248 cells with the tandem rRNA duplication. This would be consistent with finding only a small number of reads (*n*=7) spanning the tandem repeat.

To resolve this question we re-sequenced the genome of *S. iniae* QMA0248 using an ONT MinION instrument (Supplementary Information). The long reads in the nanopore assembly provided robust support for the tandem rRNA repeat at the rRNA1 locus, with >120 individual reads supporting this scaffold (Fig. S4a). In contrast, only a single nanopore read unambiguously supported the single rRNA1 locus scaffold that was well supported in the PacBio assembly (Fig. S4b). Long-range PCR using genomic DNA extracted according to the CTAB method used for ONT sequencing unambiguously supported the existence of the two variants, albeit only when up to 10× the original amount of product was visualized on a gel (Fig. S5). Thus, another factor that might have allowed better amplification and visualization of the duplicated variant is better preservation of high-molecular-weight fragments in DNA extract obtained using the CTAB method.

These findings support the idea that the original stock culture comprised a mixed population with both tandem and single rRNA1 genotypes. The sequence conservation within the tandem repeat encompasses the 16S, 23S and 5S genes of rRNA1 along with the first nine tRNA genes in the 17 gene tRNA array downstream of rRNA1 (Fig. S6). This tRNA array is unique to rRNA1 and rules out duplication via homologous recombination with a distal rRNA locus to create a large chromosomal duplication, as frequently reported in other bacteria and exemplified by the *Salmonella* model system [50, 51]. Instead, the sequence evidence is more consistent with a gene duplication and amplification (GDA) adaptive mechanism, commonly associated with antibiotic resistance and other bacterial stress responses [52]. With the recent widespread availability of long-read technologies, it has become increasingly tractable to investigate GDA and its role in genetic and phenotypic heterogeneity beyond antibiotic resistance [53]. However, further experimental work is required to confirm if the tandem repeat recurs following loss.

In *S. iniae *, we predict there has been an ancestral duplication at the rRNA1 locus with subsequent loss under certain laboratory conditions giving rise to the single rRNA1 loci scaffolds seen in the chromosome of QMA0248 (and, for example, SF1, ISET0901 and ISNO). The predominant form of the rRNA locus differed between the ONT and PacBio assemblies of QMA0248, suggesting that the addition of blood to the media in the ONT protocol may be a factor promoting selection for cells that carry the tandem repeat, and vice versa. There is a correlation between the rRNA copy number and the response rate to various substrates across diverse species [54], but the selective advantage of rRNA multiplicity within a species is less clear, with the concomitant duplication of large chromosomal segments complicating many cases of rRNA duplication [51]. An unexpected finding of this study is that *S. iniae* may be an attractive model to investigate these fundamental questions.

### Characterization of large MGEs in *S. iniae* QMA0248

**Fig. 2.**
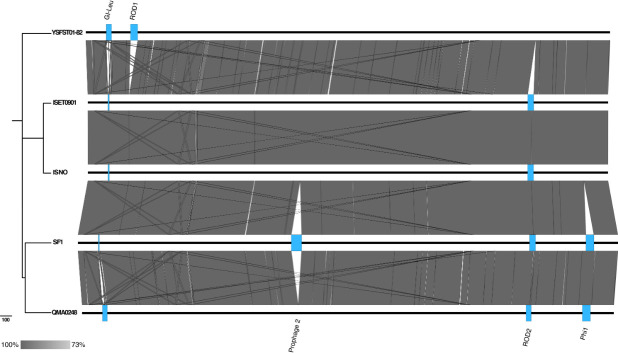
Whole-genome alignment of the five genomes QMA0248, SF1, YSFST01-82, ISET0901 and ISNO. The genomes are ordered according to their position in the core SNP-based phylogenetic tree. The maximum-likelihood (ML) phylogeny was rooted to QMA0140 (not shown) and built using 1111 SNPs. Bar, the number of substitutions represented by branch lengths. BLASTn comparison was produced using EasyFig [33] using 2000 bp as the minimum length, 50 % as the minimum identity value and 1×10^−17^ as the maximum e-value.

Our investigation of the rRNA discrepancies in *S. iniae* SF1, ISET0901 and ISNO also revealed differences in the MGE content within the available complete genomes. The chromosome of QMA0248 has a single ~12 kb genomic island (GI-Leu) inserted within the tRNA-Leu downstream of a large number of consecutive ribosomal genes (Table 2). GI-Leu encodes an integrase at its 5′ end (QMA0248_0125), which is predicted to be responsible for the GI insertion. An ~2.8 kb region at the 5′ end of the GI (87881–90708) is homologous to the fish pathogen *S. parauberis* KCTC 11537, with 90 % nucleotide sequence identity, including part of the integrase, plasmid replication gene and two hypothetical proteins. The island encodes a collagen-binding surface protein (Cna, B-type domain), which is a virulence factor with an LPxTG cell wall anchor motif, a conserved Gram-positive sorting signal [55]. An IS*30* family transposon insertion truncates the collagen-binding domain encoded by *cna*, probably leading to loss of functionality in QMA0248. This adhesin has been shown to play an important pathogenic role in *Staphylococcus aureus* by facilitating bacterial cell adherence to host collagen [56]. Most of this GI appears to have been deleted in the genomes of SF1, ISET0901 and ISNO only, along with the rRNA operon upstream of it (Fig. 2), which explains the difference with QMA0248 in total number of rRNA and tRNA genes (Table 1).

**Table 2.** Large mobile genetic elements (MGEs) and regions of difference (ROD) identified in the five *S. iniae* genomes analysed (QMA0248, SF1, YSFST01-82, ISET0901 and ISNO)

Characteristic	Genomic island (GI-leu)	ROD1	Prophage 2	ROD2	Prophage 1 (Phi1)
Coordinates*	87374–100014	177819–206359 (YSFST01-82)	848479–890501 (SF1)	1767227–1787619	1991661–2023508
Length (kb)	12.6	28.5	42.0	20.4	31.8
GC content (%)	35.9	37.2	35.3	34.3	37.6
Features	MGE; 13 tRNA and 1 rRNA operon (5S, 16S and 23S) upstream; integrase; IS*30* and IS*256* family IS elements	Integrase; IS*3* and IS*256* family IS elements	MGE; integrase	IS*3* family IS element	MGE; integrase; 1 tRNA-Cys
No. of CDSs	11	33	63	18	53
Major CDSs	Cro/CI family transcriptional regulator; ECF subfamily RNA polymerase sigma factor; plasmid replication protein; membrane protein	ESAT-6-like protein; two-component sensor histidine kinase; galactose mutarotase; 3 PTS galactitol transporter subunits (IIA, IIC and IIB)	Phage DNA replication protein; prophage antirepressor, phage capsid and scaffold protein; putative tail protein; holin; endolysin; antigen C; several phage hypothetical proteins	ESAT-6-like protein; *O*-glycosyl hydrolase; phage infection protein; 3 lipoproteins; protein kinase	DNA helicase; Cro/CI family transcriptional regulator; tail and capsid proteins; holin; lysin; DNA N-4 cytosine methyltransferase; site-specific recombinase; several phage hypothetical proteins
Best hit (% identity, % coverage)	* S. parauberis* KCTC 11537 (90, 41)	*S. dygalactiae* subsp. *equisimilis* ATCC 12394 (70, 34)	* S. parauberis* KCTC 11537 (91, 45)	* S. thermophilus* JIM 8232 (81, 13)	Bacteriophage PH10 of *Streptococcus* (71, 34)

*Coordinates are in QMA0248 GenGenBank annotation (GCA_002220115.1) unless otherwise indicated.

The genome of *S. iniae* QMA0248 harbours a single ~32 kb incomplete phage (Phi1) (1991661–2023508), including a 5′ integrase gene (QMA0248_1936), inserted upstream of a tRNA-Cys gene (Table 2). A total of 44 genes encoding phage proteins were identified, including genes involved in DNA replication such as DNA polymerase III, tail morphogenesis, as well as host lysis such as holin and lysin, in addition to 24 phage hypothetical proteins. More than half of the genes encoding phage proteins carried by QMA0248 are homologous to proteins encoded by temperate bacteriophage *Streptococcus* PH10 (56.8 % according to PHAST) [57]. Furthermore, Phi1 in QMA0248 exhibits a remarkable nucleotide sequence identity (99 %) to a prophage encoded within the SF1 genome in the same locus, whereas it is entirely absent in YSFST01-82, ISET0901 and ISNO (Fig. 2).

### Characterization of *S. iniae* QMA0248 IS

Insertion sequences (IS) were analysed in the *S. iniae* QMA0248 genome using the ISFinder database coupled with manual curation. The analysis revealed 92 IS (Table 3), which is higher than the average number per bacterial genome (*n*=38) but consistent with the lifestyle of *S. iniae* as a facultative pathogen [58, 59]. Furthermore, the number of IS found in *S. iniae* QMA0248 is substantially higher than other streptococci such as *S. mitis* strain B6 (*n*=63) but comparable to that of the Gram-positive fish pathogen *Lactococcus garvieae* [60, 61]. The 92 IS elements belong to seven different IS families and 20 IS types. These include nine novel types belonging to the IS*3*, IS*30*, IS*1182* and IS*200*/IS*605* families, which we have submitted to the ISFinder database (IS*Stin2–*IS*Stin10*) (Tables 3 and S2–S4). Around half of all IS copies in QMA0248 belong to these nine novel types, consistent with expansion of *S. iniae *-specific IS since speciation (Table 3). Amongst those genes disrupted by IS in QMA0248 is the restriction enzyme component of a type II restriction methylation system that probably recognizes ‘GCNGC’ [62]. This insertion renders the cognate MTase (QMA0248_0516) an orphan and, given the high number of GCNGC sites (3814 per Mb in QMA0248) across the genome, suggests a potential role for this MTase in global gene regulation (Table S5). By comparison, there are 7762 per Mb available GATC sites in the *Escherichia coli* K-12 MG1655 genome available for methylation by Dam, the archetypal orphan type II MTase known to play a major role in gene regulation [63]. Further discussion of the methylome data generated by PacBio sequencing of QMA0248 is provided in the Supplementary Information. Together, the *S. iniae* genome harbours a large repertoire of IS elements, which may be associated with adaptation to host or environment. Indeed, it is well accepted that IS expansion is an early sign of genome reduction as a mechanism of adaptation to the host [59, 64, 65].

**Table 3.** Summary of all insertion sequences (IS) identified in QMA0248; partial IS are suffixed by -p

IS family in QMA0248	no. of IS copies	IS types (copy no., mean % amino acid identity)
IS*3*	32	IS*Sag2* (10, 98.6), IS*Sag2*-p (2, 98.7) IS*981* (8, 99.8) IS*Spy1* (1, 86.7), IS*Spy1*-p (1, 90.5) *IS*Stin6* (3, 94.5), *IS*Stin7* (4, 90.0), *IS*Stin5* (2, 69.3), unclassified most similar to *IS*Stin5-p* (1, 73.8)
IS*30*	22	IS*Sag9* (3, 99.6), IS*Sag9*-p (5, 99.6) *IS*Stin4* (2, 93.3), *IS*Stin4*-p (2, 89.6), *IS*Stin9* (9, 81.4), *IS*Stin2* (1, 84.7)
IS*256*	17	IS*Stin1* (16, 91.0), unclassified most similar to IS*Stin1*-p (1, 90.2)
IS*1182*	13	*IS*Stin8* (7, 86.3), *IS*Stin8*-p (2, 89.8), *IS*Stin3* (1, 87.7), *IS*Stin3*-p (2, 87.1), unclassified most similar to *IS*Stin8-p* (1, 70.5)
IS*200*/IS*605*	5	*IS*Stin10* (3, 99), *IS*Stin10-*p (1, 98.6), unclassified most similar to *IS*Stin10* (1, 86.5)
IS*L3*	1	Unclassified most similar to IS*Sth1*-p (1, 77.8)
IS*110*	2	Unclassified most similar to IS*L4* (2, 65.8)

*Novel IS element.

### Phylogenetic and comparative analysis of *S. iniae* QMA0248, SF1, YSFST01-82, ISET0901 and ISNO

**Fig. 3.**
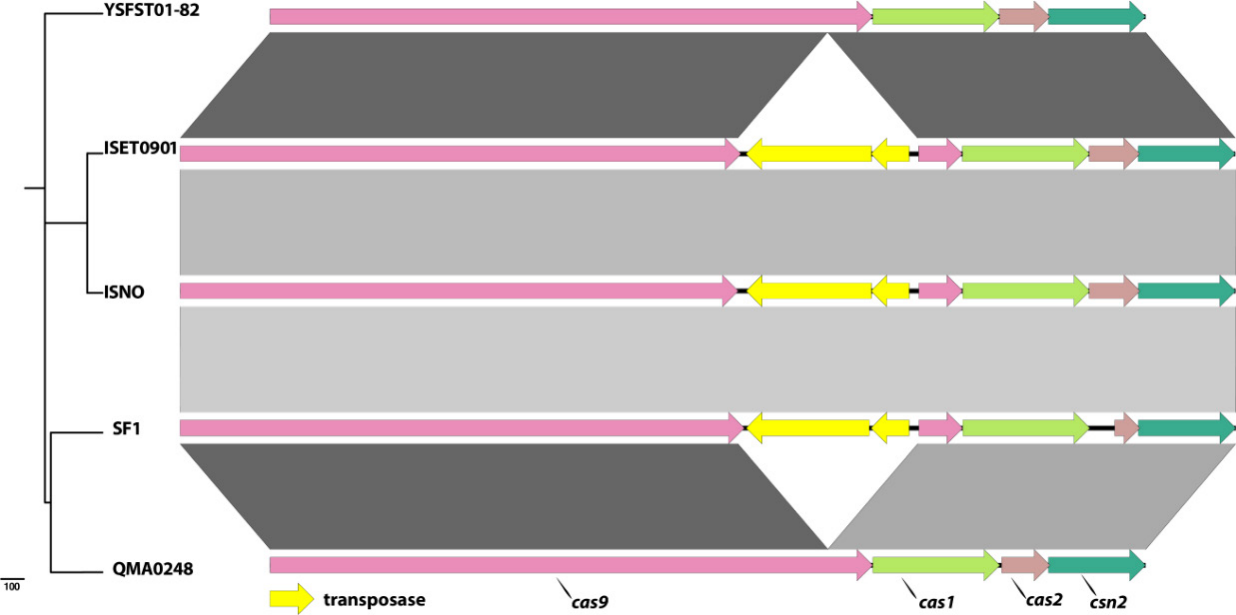
Comparison of the CRISPR/Cas region between QMA0248, SF1, YSFST01-82, ISET0901 and ISNO. Alignment of Cas genes where the genomes are ordered according to their position in the phylogenetic tree (left). The maximum-likelihood (ML) phylogeny was rooted to QMA0140 (not shown) and built using 1111 SNPs. The scale bar indicates the number of substitutions represented by branch lengths. Arrows correspond to Cas genes, which are labelled at the bottom. Figure was produced using EasyFig [33] using 500 bp as the minimum length, 90 % as the minimum identity value and 0.001 as the maximum e-value.

The core genome of *S. iniae* QMA0248, SF1, YSFST01-82, ISET0901 and ISNO accounts for ~75 % of the chromosome. IS were compared between the reference chromosome of QMA0248 and each of the *S. iniae* chromosomes (SF1, YSFST01-82, ISET0901 and ISNO) using ACT [34]. Although IS elements typically result in genomic rearrangements and loss of synteny, this is not seen in *S. iniae *. This lack of rearrangement is reflected by the consistent pattern of GC skew in the genome of *S. iniae* QMA0248 (Fig. 1). Eight IS copies out of the 92 detected in QMA0248 are absent in the genomes of SF1, ISET0901 and ISNO only (Table S2). Other IS elements are unique to the genomes of SF1, ISET0901 and ISNO in syntenic positions. An interesting example is an IS*981* (SF1 locus_tag: K710_0799 and K710_0800), inserted in the *cas9* gene in the CRISPR/Cas region only in those three genomes (Fig. 3). Another example is IS*Stin1*, inserted only in SF1, ISET0901 and ISNO (SF1 locus_tag: K710_0761). Additionally, three IS copies are present in syntenic positions only in QMA0248, ISET0901 and ISNO. Eight insertions are absent in YSFST01-82 only and another seven IS copies are found exclusive to QMA0248 (Table S2).

Our comparative analyses suggest that one or more of the *S. iniae* genomes under comparison have been misassembled. Evidence for this conclusion is found in the observed pattern of MGE conservation. In addition to the IS differences, the five *S. iniae* genomes have major differences in the content of other MGEs that reflect variations in the length of their respective chromosomes (Table 1). These MGEs include two prophages (Phi1 and Phi2), a GI and two other ROD (Table 2, Fig. 2). This includes an ~28 kb region that is only found in YSFST01-82 (ROD1), and an ~20 kb region that is present in four genomes but almost entirely absent from YSFST01-82 (ROD2) (Table 2, Fig. 2).

Most variations in MGEs (including IS) were found to be incongruent with the core SNP phylogeny. For instance, the deleted ~12 kb GI-Leu and the *cas9* gene disrupted by IS*981* exist only in SF1, ISET0901 and ISNO (Figs 2 and 3), but these three isolates appear on divergent branches, indicating potential independent events (Fig. S7). To investigate the discrepancies between MGEs and phylogeny we compared multiple phylogenetic trees that were reconstructed using different methods, including the core genome, core SNP and using different software (Fig. S7). All phylogenies consistently revealed that *S. iniae* isolates QMA0248 and SF1 cluster together in one clade, whereas ISET0901 and ISNO cluster in another, and all four isolates cluster separately to YSFST01-82, the latter diverging earliest from the root (Fig. S7).

### Discrepancies between the *S. iniae* genomes are probably due to misassembly

The genomes of ISET0901 and ISNO were both assembled from Illumina data using BioNumerics (Applied Math) with the genome of *S. iniae* SF1 as a reference [12, 13]. *S. iniae* SF1 was assembled *de novo* from a combination of 454 GS FLX+, Illumina MiSeq and Sanger sequencing [12]. During the preparation of the present paper, SF1 was removed from the NCBI RefSeq database. YSFST01-82 was also a hybrid assembly (454 GS FLX Titanium, Opgen optical mapping and Sanger sequencing) but it remains in the RefSeq database and is the designated representative genome for *S. iniae* at NCBI (https://www.ncbi.nlm.nih.gov/genome/?term=streptococcus+iniae; accessed 10 November 2020)

We have no reason to suspect that the YSFST01-82 genome assembly is inferior to that of QMA0248, but adopting the latter as an alternative representative *S. iniae* genome is justified for investigators wishing to take advantage of a manually curated set of MGEs. In contrast, we strongly recommend not using ISET0901 or ISNO in future comparative studies of *S. iniae* genomes. Reference-guided assembly was introduced to enable comparisons between two very closely related isolates. However, this practice can result in the erroneous inclusion of MGEs that exist in the template genome but are absent from the comparison strain. Even with careful curation it is impossible to avoid misplacing repetitive sequences such as IS, as observed here in the case of *cas9* insertion and the other eight IS copies that are absent in SF1, ISET0901 and ISNO only. Moreover, reference-guided assemblies may result in the loss of novel regions that are only present in the newly sequenced strain, in which case a *de novo* approach is always required [66]. Although reference-guided assembly is no longer generally accepted for prokaryote genomes, a number of examples remain available in public repositories such as GenBank. For both ISET0901 and ISNO the assembly strategy is clearly outlined in the comment field of the GenBank file, and in the primary publications [12, 13]. Nevertheless, the consequences of using such genomes in downstream analyses may not be apparent to all [e.g. all three genomes are available in widely used genome databases such as PATRIC (www.patricbrc.org version 3.5.36) [67].

Removal of some early hybrid 454 complete genomes from public repositories such as RefSeq should help maintain the quality of available complete genomes. Long-read sequencing data from Pacific Biosciences and Oxford Nanopore Technologies bring complete bacterial genomes within reach of most laboratories, but here also significant care is often required to avoid misassembly. Furthermore, as illustrated here and in other studies [68, 69], what appear to be misassemblies may in fact be biologically relevant. Ultimately the onus is on the user of public data to exercise caution when validating the source, assembly strategy and quality of available complete genomes.

## Conclusions

We assembled and annotated a high-quality complete genome sequence for *S. iniae* QMA0248, including manual curation of 92 IS. Comparative analysis with publicly available complete genomes of *S. iniae* SF1, YSFST01-82, ISET0901 and ISNO revealed discrepancies in the MGE content consistent with errors introduced by reference-guided assembly of ISNO and ISET0901. Such problems are not new, but many bacterial genomes assembled in this way remain in public repositories of complete genomes. Our results emphasize the need to critically appraise complete genome assemblies prior to comparative analysis. Despite long-read sequencing becoming the gold standard for complete genome assembly of bacterial isolates, caution is needed to avoid misassembly. Long-read sequencing can also characterize heterogeneity within cultures that may be biologically relevant but intractable by other approaches. To better understand how IS, GIs and other mobile elements contribute to *S. iniae* diversity, there is a need for larger genomic studies using global collections of *S. iniae* isolates from dissimilar origins. The genome of *S. iniae* QMA0248 represents an important resource for future *S. iniae* comparative genomic and evolutionary studies.

## Supplementary Data

Supplementary material 1

Supplementary material 2
