# Supplementary material for: A high-quality reference genome for the fish pathogen Streptococcus iniae
Source: Microb Genom. 2022 Mar 1;8(3):000777. doi: 10.1099/mgen.0.000777 (PMC9176272; doi:10.1099/mgen.0.000777)
Supplement: Supplementary material 1 [file mgen-8-0777-s001.pdf]

Supplementary Information

A high quality reference genome for the fish pathogen *Streptococcus iniae*

Areej S. Alsheikh-Hussain[1,2], Nouri L. Ben Zakour[1,2,3], Brian M. Forde[1,2], Oleksandra Silayeva[4], Andrew C. Barnes[4], and Scott A. Beatson*[1,2].

[1]School of Chemistry & Molecular Biosciences, [2]Australian Infectious Diseases Research Centre and [4]School of Biological Science, The University of Queensland, Brisbane, Queensland, Australia;

Present address: [3]The Westmead Institute for Medical Research and the University of Sydney, Sydney, New South Wales, Australia

* Corresponding authors:

Scott Beatson: s.beatson@uq.edu.au

Andrew Barnes: a.barnes@uq.edu.au

S. iniae QMA0248 methylome

DNA methylation guides numerous critical processes including defence against foreign DNA, DNA replication and repair, gene expression and virulence. Analysis of PacBio sequence data enabled the detection of genome-wide DNA methylation. Three DNA methyltransferases (MTases) were encoded in the QMA0248 genome. On the basis of homology to known MTases, QMA0248_0514 (annotated as M.Sin248ORF514P in REBASE) likely targets the GCNGC motif and QMA0248_1949 (annotated as M.Sin248ORF1949P in REBASE) likely targets GCCHR (1). QMA0248_0505 (annotated as M.Sin248ORF0505P in REBASE) is encoded ~5kb upstream of QMA0248_0514 but has no close functional homologs and thus has an unknown recognition sequence (1). A small fraction of GCNGC and GCCHR motifs were methylated in QMA0248 (<3%). Collectively these two motifs account for 21,421 sites across the chromosome (or 1 every 100 bp) suggesting a potential role for methylation in the regulation of gene expression in QMA0248. This figure is roughly equivalent to the frequency of Dam GATC sites in *Escherichia coli*. Further work is required to determine if the activity detected here is biologically meaningful.

5-methylcytosine DNA methyltransferase QMA0248_0514

A putative Type II 5-methylcytosine (m5C) DNA MTase (QMA0248_0514, annotated as M.Sin248ORF514P in REBASE) likely targets the GCNGC motif (1). Uncharacterised homologs with 99% amino acid identity are found in 8 other available *S. iniae* complete or draft genomes including SF1, YSFST01-82, ISET0901, ISNO and 89353 (1). In most cases restriction enzymes predicted to recognise GCNGC are predicted to be encoded nearby, or immediately adjacent to

the respective MTase gene. Notably, in QMA0248 the adjacent restriction enzyme (QMA0248_0515) is a pseudogene that has been truncated by an IS*981*. In *S. iniae* KCTC 11634BP, the orthologous gene in its draft quality 454 genome was truncated the same point by a contig break (2), suggesting that in both QMA0248 and KCTC 11634BP the MTase does not function as part of a restriction-modification system. The GCNGC motif is found 8074 times in the QMA0248 genome suggesting that methylation activity could have wide-ranging regulatory consequences.

The closest homologs to QMA0248_0514 for which MTase activity has been determined is the M.CmaLM2II enzyme from *Clostridium mangenotii* LM2 and the M.LmoJ3I enzyme from *Listeria monocytogenes* J3115. Despite sharing modest overall amino acid similarity to QMA0248_0514 (59% and 45%, respectively), regions of high amino acid identity within their predicted target recognition domains (34/34 for M.CmaLM2II and 32/24 for M.LmoJ3I) support the prediction that QMA0248_0514 would also methylate the 2nd cytosine of the GCNGC motif. Detection of m5C using PacBio data is normally unreliable (3). As expected, methylation was detected at only a small fraction of GCNGC sites in the QMA0248 genome and the consensus motif determined by the PacBioSMRT-Portal software includes additional bases that are probably artefactual (e.g. GCNGCAGC) (Supplementary Table S5). Further experimental work (such as using Tet1 pre-treatment to enhance detection of m5C with PacBio sequencing, or Oxford Nanopore sequencing) is needed to determine the true extent of cytosine methylation in the *S. iniae* genome and its role in gene regulation.

N4-methylcytosine DNA methyltransferase QMA0248_1949

The second *S. iniae* QMA0248 MTase (QMA0248_1949, known as M.Sin248ORF1949P in REBASE) shares 95% amino acid identity with the Orphan (gamma) N4-methylcytosine (m4C) DNA MTase M.NgoDCXV (gb|AJ004687.2) from *Neisseria gonorrhoeae*, which specifically targets the GCCHR motif. M.NgoDCXV homologs are remarkably rare and confined to a few streptococcal species (including *S. iniaie* SF1). No specific methylation of GCCHR was detected in the QMA0248 genome but 126 motifs that partially overlapped with GCCHR showed evidence of methylation (Supplementary Table S5). The GCCHR motif is present in 13,347 locations in the QMA0248 genome so this represents only a fraction of available sites. The m4C modification is normally detectable from PacBio sequence data, therefore further work is required to determine if QMA0248_1949 is expressed and functional.

Putative Type II N4-methylcytosine or N6-methyladenine DNA methyltransferase QMA0248_0505

The third MTase in *S. iniae* QMA0248 (QMA0248_0505, known as M.Sin248ORF0505P in REBASE) is encoded ~5kb upstream of QMA0248_0514 and shares a similar strain distribution. There are no close homologs of QMA0248_0505 for which a recognition site has been determined. Accordingly it has been annotated by REBASE as a putative Type II N4-cytosine or N6-adenine DNA methyltransferase of unknown recognition sequence (1).

Supplementary Figures

**YSFST01-82**	1	2	3	4	5	6	7	8	15	16	17	18		
**ISET0901**	1*	2	3	4*	19	15								
**ISNO**	1*	2	3	4*	19	15								
**SF1**	1	2	3	4	5	6	19	15						
**QMA0248**	1	2	3	4	5	6	7	8	9	10	11	12	13	14

Figure S1: Graphical representation of spacers in CRISPR between YSFST01-82, ISET0901, ISNO, SF1, and QMA0248. Each box represents a spacer, where the same colour and number indicate identical spacers. *: A spacer with an additional direct repeat sequence.

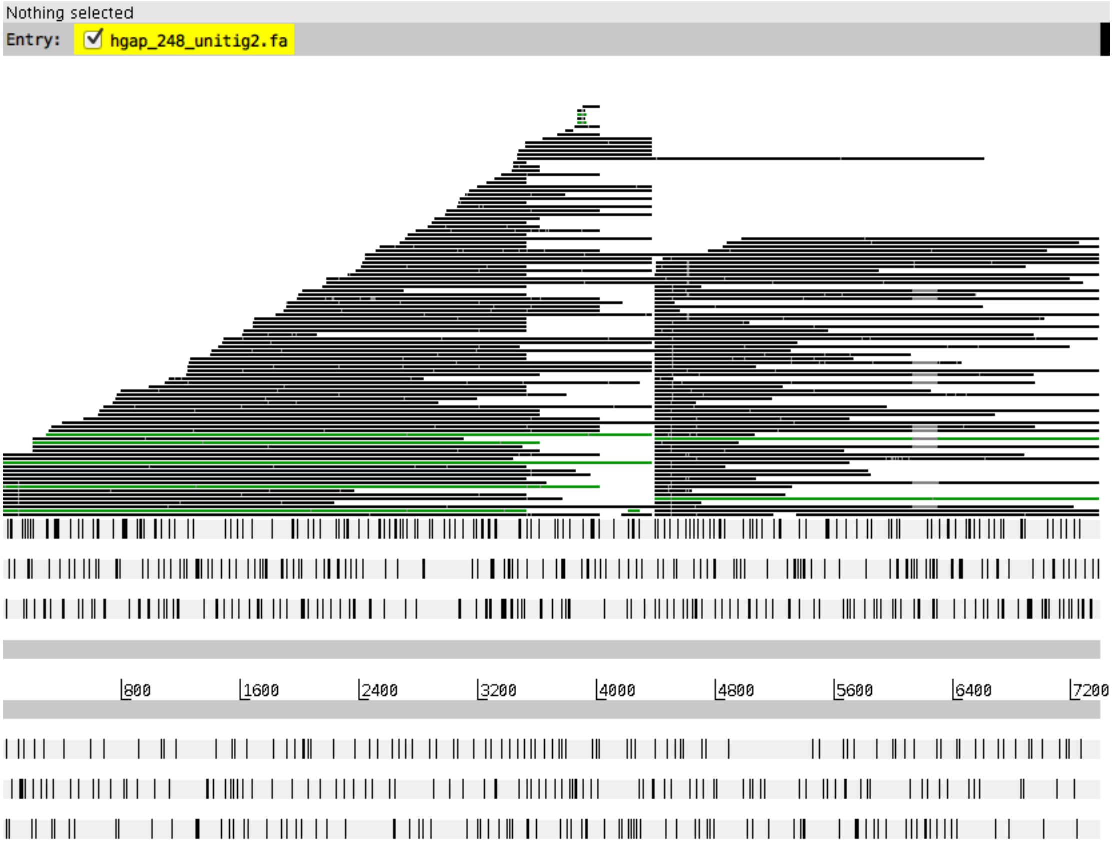

Figure S2: PacBio raw reads pileup of *Streptococcus iniae* QMA0248 on the spurious 7.2 Kb rRNA contig. Alignment of reads is visualised using BamView implemented in Artemis. Horizontal lines on the upper panel correspond to the aligned reads. Duplicate reads are coloured green and other reads are coloured black. The lower panel corresponds to the forward and reverse strands of the 7.2 Kb contig, where vertical black lines indicate stop codons.

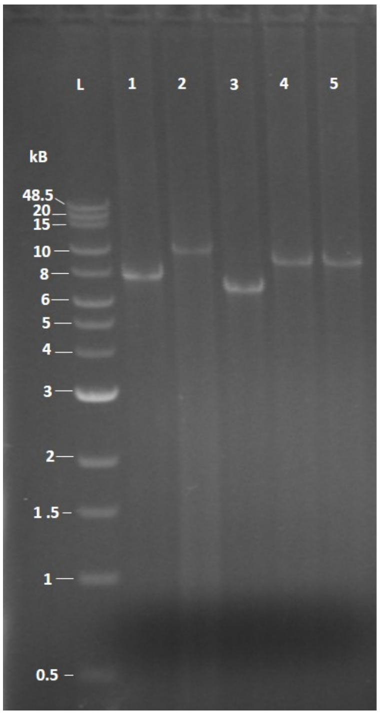

Figure S3: Long-range PCR across rRNA operons. Gel electrophoresis of long range PCR products across five rRNA operons of *S. iniae* QMA0248 (lanes 1-5 in operon-wise order as listed in Table S6, rRNA1 to rRNA5). Predicted PCR produce size: rRNA1 (7578 bp), rRNA2 (9995 bp), rRNA3 (6695 bp), rRNA4 (8709 bp), rRNA5 (8722 bp). Primers are listed in Table S6. "L" denotes 1 Kb Extend DNA Ladder (NEB).

A

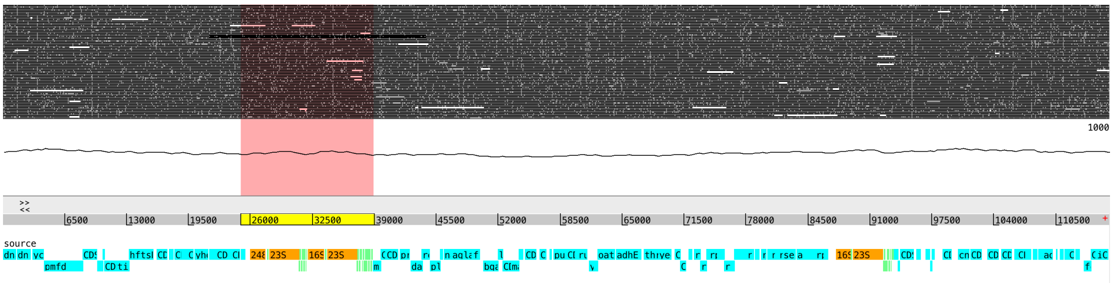

B

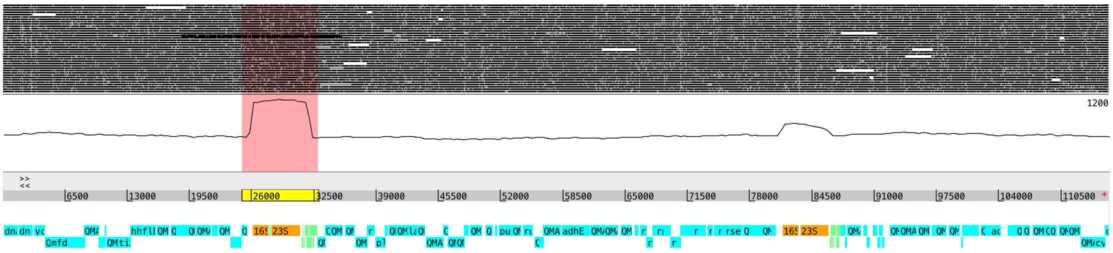

Figure S4: *Streptococcus iniae* QMA0248 nanopore assemblies. QMA0248 genome viewed in Artemis with rRNA1 locus highlighted in red vertically across all panels. Top panels shows alignment of nanopore reads mapped to either the genome containing **(A)** tandem rRNA repeat locus or **(B)** the single rRNA locus. Reads visualised using BamView with a single spanning read highlighted in black as an example. Middle panels show read coverage as graph with scale on top right hand corner of panel. Bottom panel shows annotated sequence numbered from nucleotide 1 on left hand side. Coding sequences are coloured aqua, rRNA genes are coloured orange and tRNA genes are coloured green.

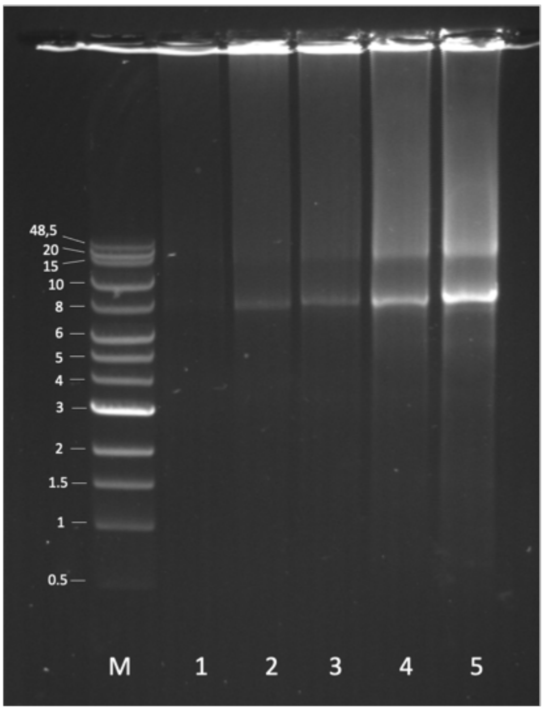

Figure S5: Long-range PCR using CTAB genomic DNA extraction protocol. As PCR had previously only amplified a single copy at the rRNA1 locus, the reaction was re-run under previously used conditions except DNA extracted using CTAB method was provided as a template (the extraction used for ONT library preparation). This time PCR unambiguously supported the existence of two variants, but only when larger amounts (over 10 μL) of PCR products were run on the gel (lanes 4 and 5). However, when smaller loads (under 5 μL) of PCR product were run only a single copy of the locus was visualised clearly and the tandem repeat rRNA variant amplicon was very faint (lanes 2 and 3). “M” denotes 1 Kb Extend DNA Ladder (NEB).

A

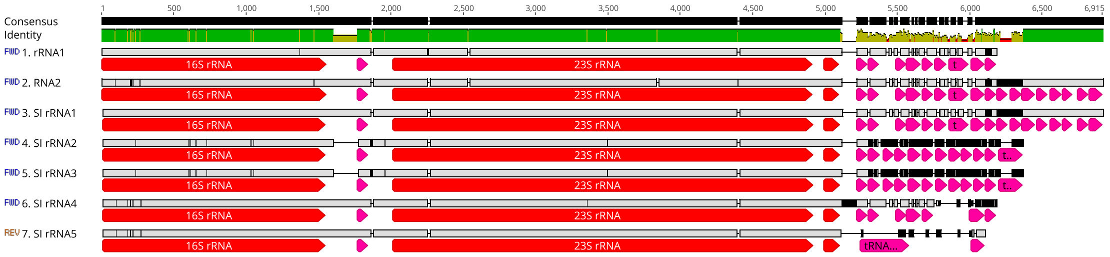

B

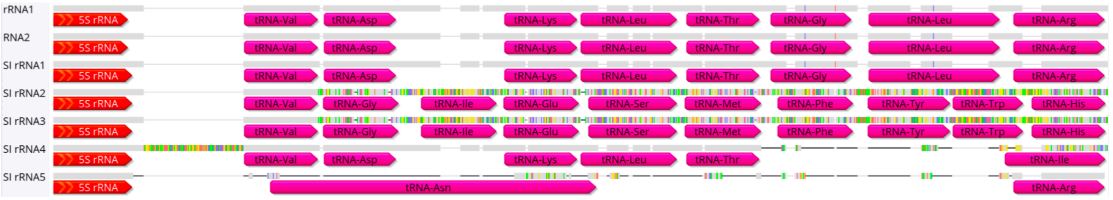

Figure S6: Alignment of rRNA loci in *Streptococcus iniae* QMA0248. Schematic diagrams of the rRNA loci from *S. iniae* QMA0248 aligned at the nucleotide level with annotations coloured by feature type (red: rRNA gene; pink: tRNA gene). Complete loci are shown in panel **(A)** with a zoomed view shown in panel **(B)** to enable tRNA gene labels to be visible. Labels on the left refer to repeat 1 (rRNA1) and 2 (rRNA1) of the tandem rRNA repeat locus assembled in the ONT assembly or SI rRNA1-5 referring to the five rRNA regions from the *S. iniae* QMA0248 complete PacBio genome. Figure shows that the tRNA array downstream of rRNA1, rRNA2 and SI rRNA1 does not match any other rRNA locus, consistant with duplication at SI rRNA1. In the ONT assembly, The rRNA repeats are arranged with a 155 nucleotide spacer between the 3' end of tRNA-Pro of the upstream repeat and the 5' end of the 16S gene of the downstream repeat. Figures were prepared in Geneious Prime V2021.2.2 to scale (nucleotide scale is top of panel A). Note that as this is a gapped nucleotide alignment some open reading frames have been stretched because of long gapped regions in the tRNA array (e.g. tRNA-Asn in SI rRNA6).

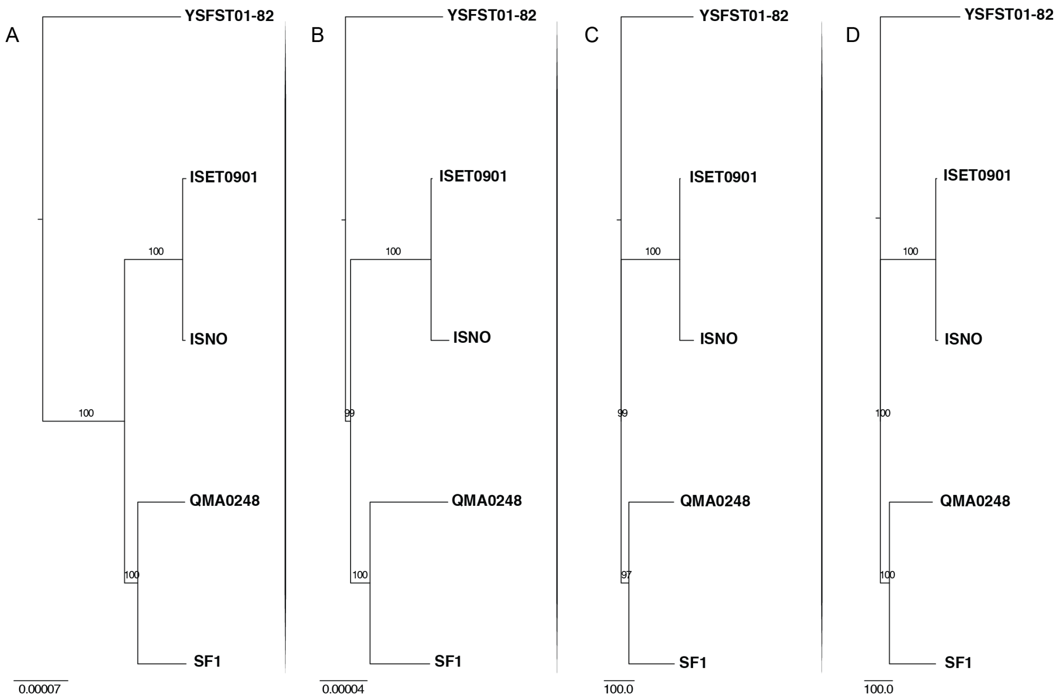

Figure S7: Alternative tree-building methods. Phylogenetic trees constructed for *S. iniae* based on core genome using Mauve (A) and Parsnp (B), and core SNP using Nesoni (C) and Snippy (D). Maximum likelihood (ML) phylograms were built using RAxML where bootstrap support values are shown on branches. Trees are rooted using QMA0140 (not shown). Branch lengths correspond to the number of substitutions (C and D), and the number of substitutions per site (A and B). ML phylograms in A and B were built from 1,928,667 bp and 1,856,820 bp core genome alignment, respectively, whereas C and D were built using 1,241 and 1,116 substitutions, respectively.

1. Roberts RJ, Vincze T, Posfai J, Macelis D. REBASE—a database for DNA restriction and modification: enzymes, genes and genomes. Nucleic acids research. 2009;38(suppl_1):D234-D6.

2. Choi HS, Kwon MG, Kim MS, Park MA, Kim D-W, Park J-Y, et al. Draft genome sequence of beta-hemolytic Streptococcus iniae KCTC 11634. Genome announcements. 2013;1(6):e00897-13.

3. Clark TA, Lu X, Luong K, Dai Q, Boitano M, Turner SW, et al. Enhanced 5-methylcytosine detection in single-molecule, real-time sequencing via Tet1 oxidation. BMC biology. 2013;11(1):4.
